# The State of Research in Fracture-Related Infection—A Bibliometric Analysis

**DOI:** 10.3390/medicina58091170

**Published:** 2022-08-29

**Authors:** Nike Walter, Nicolás Orbenes, Markus Rupp, Volker Alt

**Affiliations:** 1 Department for Trauma Surgery, University Medical Center Regensburg, 93053 Regensburg, Germany; 2 Department of Trauma Surgery, Klinikum Rechts der Isar, Technical University of Munich, 80333 Munich, Germany

**Keywords:** fracture-related infection, bibliometry, research scores, medical information science

## Abstract

*Background and Objectives*: Fracture-related infection (FRI) is a challenging complication in trauma surgery. A consensus definition of FRI has only recently been published. Therefore, the purpose of this study was to evaluate the state of research related to FRI. *Material and Methods:* A systemic literature review was conducted on research on FRI published between 2017 and 2020. The Web of Science database was used, and a bibliometric analysis was performed. To provide robust evidence regarding the impact of publications, the behavior of publications in non-traditional dissemination channels was analyzed. For this, the Research Interest Score and the Altmetric Score were combined. The Research Interest Score was calculated from information extracted from ResearchGate, while Altmetric Score includes information from different websites and apps with a significant volume of traffic, such as Twitter. *Results:* A total of 131 published papers were identified. The most significant contribution came from the United States and European countries. The most relevant articles were published by the journal *Injury—International Journal of the Care of the Injured*. A positive correlation was observed between the number of citations and Research Interest Scores, whereas the number of citations and Altmetric Score showed no correlation. The social media platform most used by FRI researchers was Twitter. *Conclusions:* By evaluating the status of publications for FRI between 2017 and 2020, an upward trend in the number of publications was evident. This could be related to the increasing acceptance of the long-needed definition for FRI and the implications it carries for daily clinical practice.

## 1. Introduction

Fracture-related infection (FRI) is a major complication in musculoskeletal trauma surgery. Over recent years, the incidence of fractures as well as rates of subsequent infections increased, especially in elderly patients indicating a potential challenge for stakeholders in the health care system [1,2]. FRI is associated with prolonged length of hospital stay, heightened healthcare cost, and a significant deterioration of patients’ quality of life [3,4,5]. For all these reasons, it is essential to devise new treatment strategies, prevention methods, and implement interdisciplinary approaches to FRI [6,7]. However, an essential prerequisite for correct diagnostics and most ideal therapy is precise terminology. For FRI, a variety of terms such as posttraumatic osteomyelitis or infected non-union can be found in the literature and clear definition and, hence, clarity concerning treatment algorithms has been lacking for this entity [8,9,10]. Therefore, a consensus group definition was developed in 2017 including confirmatory and suggestive diagnosis criteria [11]. Since then, the research spanned a wide range of topics, including the value of different diagnostic biomarkers and techniques [12], the etiology of causative pathogens and their antibiotic susceptibility [13,14,15] up to novel in vivo models to study biofilm formation and the potential of new therapies such as bacteriophages [16,17,18].

Due to the large number of publications and continuing advances, it is important to determine which are suitable for use in clinical decision making. A commonly used method for evaluating the level of influence of a publication is the number of citations. In the last decade, there has been an effort to identify the most relevant articles in medical specialties through identification of the most cited works in the field [19]. Bibliometric analysis is a statistical method that allows assessing the development trends and characteristics of a given research topic based on published research over a given period [20]. Bibliometric analysis has previously been used in diverse orthopedic pathologies, including periprosthetic joint infections (PJI) and FRI [21,22]. This study aims at providing a comprehensive overview of the current state of the literature.

## 2. Materials and Methods

### 2.1. Search Strategy

Data were retrieved from the Web of Science (WoS) electronic database (SCI-Expanded), which is designed for bibliometric analysis. WOS covers approximately 34,000 journals with over 75 million records, includes the multiple sub-databases and provides a basis for the Thomas Reuter impact factor [23].

The search algorithm included the following keywords: fracture-related infection, OR infected nonunion, OR posttraumatic osteomyelitis OR implant-associated infection. A search for articles published between 1 January 2017 and 31 December 2020 was performed. As this study was performed using global research, there were no language restrictions.

### 2.2. Data Collection

**Figure 1:**
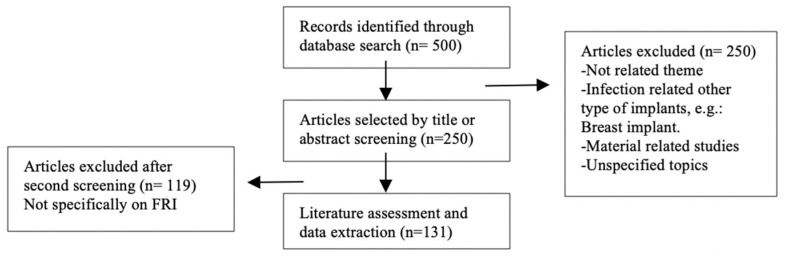
Flowchart of the identification of relevant articles.

In total, 500 publications were identified. A screening was performed to validate that the articles referred to the field of FRI. Subsequently, 250 were excluded after evaluation of the title and abstract. Additionally, 119 publications were excluded after full article screening (Figure 1). The data were extracted in June 2021 from the Web of Science and exported to plain text. The data were then tabulated in a spreadsheet using Microsoft Excel (Microsoft, Redmond, WA, USA). The criteria evaluated were the year of publication, the total number of citations, number of citations per year, institution, authors, the journal published, subject, and country of origin. Firstly, the Research Interest Score was searched by digital object identifier (DOI) number or by title in ResearchGate (http://www.researchgate.net/, accessed on 2 January 2021). Consequently, the Altmetric Score was determined using the Altmetric bookmarklet (https://www.altmetric.com, accessed on 2 January 2021). The latter was used to assess social media behavior. Only the social networks Facebook and Twitter were evaluated due to the limitations of the Altmetric bookmarklet. In addition, to analyze the correlation between the different scores, a multivariate linear regression using ordinary least squares (OLS) was performed in which the following four variables were compared and analyzed: the number of citations, the Research Interest Score, the Altmetric Score, and the year of publication.

Finally, the 10 most cited studies were evaluated in detail, using the same criteria mentioned above.

This is an observational study. The Research Ethics Committee of the University Hospital Regensburg has confirmed that no ethical approval is required.

## 3. Results

**Figure 2:**
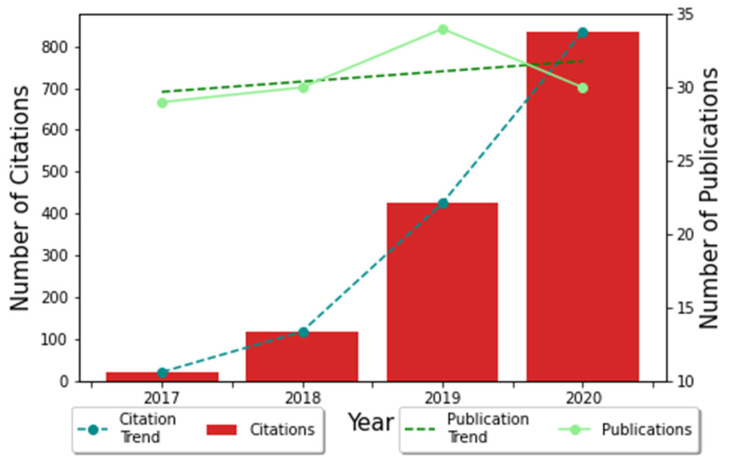
The total annual number and citations of publications between 2017 and 2020.

A total of *n* = 131 articles on FRI were collected from the WoS. Most of these articles were in English (94%), followed by German. The total number of citations of these articles was 1895. The number of publications and citations peaked in 2020. From 2017 to 2020, global publications and total citations have shown a progressive upward trend, even doubling in number between 2019 and 2020 (Figure 2). The increasing trend was also maintained in the last 18 months (January 2021 to August 2022) yielding *n* = 106 additional publications with *n* = 194 citations. In parallel, it was observed that research is mainly focused on prevention, diagnosis, and treatment.

### 3.1. Countries

Publications came from 24 countries, half of them from Europe (*n* = 13), followed by Asia (*n* = 4). The country with the most publications in Asia was China; in Africa, it was Egypt; and in Europe it was Germany. From 2017 to 2020, the United States had the highest number of publications (*n* = 30), followed by Germany (*n* = 27) and Switzerland (*n* = 22). It is essential to consider that many of the papers were multi-centric and carried out by various working teams. In this sense, to organize the origin of the papers, the most significant number of authors of a site was considered and, in case of equality, the origin of the primary author.

### 3.2. Authors

The author with the highest number of publications was WJ Metsemakers, followed by MA McNally (19) and M Morgenstern (15). All authors in the top 5 in terms of the number of publications were from the top 5 institutions (KU Leuven, AO Research Institute Davos, University of Basel, Oxford University Hospitals, and Charite Berlin). There was significant heterogeneity in the origin of the authors, with most of them coming from Europe and the USA.

### 3.3. Journals

Articles were published in 66 different journals. *Injury—International Journal of the Care of the Injured* (13.5%) had the highest number of publications, followed by *The Journal of Orthopedic Research* (6.7%). Third on the list was *Journal of Orthopedic Trauma* (4.5%) (Table 1).

### 3.4. Top 10 Most Cited Publications

The 10 most cited articles on FRI were cited between 20 and 268 times by June 2021. These 10 articles alone accounted for 47.7% of the citations (*n* = 895). The most cited article was a study by Bingyun Li in 2018 [24]. The second most cited article was one by Lei Tan et al. in 2018 [25], and the third was by an expert group consensus [11]. All articles were published in English. Finally, when analyzing our multivariate linear regression, we could see that Research Interest positively affects the number of citations being significant at any confidence level. Contrastingly, the Altmetric Score does not positively affect the number of citations. The year of publication weighs on the total number of citations of an article, reflecting the fact that the newer an article is, the less exposure it has had and, therefore, the fewer times it has been cited (Table 2).

## 4. Discussion

Between 2017 and 2019, an initial upward trend in publications was observed; however, this reached a plateau, maintaining in 2020 several publications as in previous years. In addition, when analyzing the number of citations, it was observed that these grew each year significantly. This increase may be due to the growing acceptance and use of the formal definition of FRI. By having an established working definition, research and clinical utilization could rise.

Articles in this field came from 24 countries. The academics who have most influenced FRI research are those from the United States and Europe. Among the top 10 authors, 6 were orthopedic surgeons, and 4 were experts in infectious diseases, demonstrating a multidisciplinary approach to FRI treatment.

The publications with the highest number of citations came from different backgrounds related to the management of this complex pathology. The first discusses the rise of antibiotic resistance and the formation of super-resistant strains and our challenges and opportunities in managing this issue [24]. The second publication relates to new strategies to eradicate biofilms [25], and lastly, there is the already named consensus of 2018, which delivers guidelines and definitions to be able manage FRI [11].

It is important to note that although these are the publications with the most citations, the level of evidence does not appear to be the determining factor in terms of the number of citations. Previous studies have reported that higher levels of evidence should be more compelling for orthopedists trying to solve clinical problems [32]; however, clinical relevance seems to play a critical role in the number of citations of a publication. For instance, the publication of an expert opinion with a level of evidence V, is one of the most cited [11]. The increasing number of papers leads us to suspect that there will be publications with stronger evidence in the most cited articles in the future.

The top 10 FRI research articles were collected and analyzed for relationships between citations, year of publication, and Research Interest and Altmetric Scores. Most of the publications at the top of the list were published in *Injury—International Journal of the Care of the Injured* and *Bone & Joint Journal*.

The 10 most-cited publications were predominantly published in orthopedic journals, although not exclusively. It is important to emphasize that in this list, only *Biomaterials*, with two publications within these 10, has an impact factor that places it within the 500 most relevant journals in 2021 (Journal Citation Reports by Web of Science). It should be noted that of the 10 most-cited publications, 70% are in Open Access. As has been seen in previous studies, this fact increases the number of citations independently [33]. The Altmetric Score and Research Interest Score are two different tools used to evaluate the literature [34]. The scope of the Altmetric Score is much broader than that of the Research Interest Score, with more than 16 weighted composite scores from websites such as Twitter and Facebook. In contrast, the Research Interest Score is calculated from ResearchGate, a for-profit scientific networking and collaboration website with features similar to other social networking sites [35]. The site creates profiles with information gathered from bibliographic databases and other sources and allows researchers to create profiles by registering on the site.

A negative correlation was observed between the Altmetric Score and the number of citations; however, the results are somewhat controversial. Previous studies have shown a null correlation between the number of citations and the Altmetric Score [36,37]. In contrast, several studies have shown a weak positive correlation between the number of citations and the Altmetric Score in highly cited articles. Altmetric, or alternative metrics, is a new score that seeks to determine the attention a publication attracts by tracking and measuring online mentions, whether on blogs or social media platforms [38]. This score could be used as a complementary tool to help evaluate articles rather than replacing the number of citations [39]. From the Altmetric Score information, it was found that most researchers use Twitter instead of Facebook to disseminate knowledge. A study from 2019 also showed that Twitter is more popular than Facebook for scholarly communication [40]. In contrast to the Altmetric Score, the present study found a positive correlation between citation count and Research Interest Score. This is a valuable complement to assess academic impact based on citations; however, more research is needed to confirm this relationship [41].

The main limitation of this study is that the bibliometric analysis was solely based on the Web of Science. The Web of Science covers approximately 34,000 journals with over 75 million records including the sub-databases Science Citation Index, Social Sciences Citation Index, Arts and Humanities Citation Index, Conference Proceedings Citation Index, Book Citation Index, and Emerging Sources Citation Index [24]. Further, the Thomas Scientific impact factor is based on the Web of Science database [34]. In addition, the Web of Science is older than the Scopus database, which was launched by Elsevier Science in 2004 and provides better graphical rankings of the citation analysis and is more detailed than the citation analysis of Scopus [42]. However, the Web of Science queries the citation, abstract, and keyword identifiers, but one must acknowledge that articles mentioning the keywords of interest in the methods sections are omitted, and thus, completeness of the identified list of articles cannot be fully ensured.

As fracture-related infection is a broad topic relevant to numerous clinical specialties, it was decided to include in vitro and animal studies. In addition, the Altmetric and Research Interest Score results were a combination of multiple indicators, with the ratio of the number of times cited to the total number of Altmetric and Research Interest Scores analyzed. However, while detailed scoring could provide relevant information between citations and the score for each indicator, this was not undertaken in the present study. Some websites protect their content behind login pages, preventing Altmetric from accessing all their data. Therefore, in some cases, it is not possible to obtain the information.

In conclusion, between 2017 and 2020, there is an upward trend in publications and citations related to FRI. The significant increase may be due to the increasing use of the definition of fracture-related infections. We believe that the number of citations, the number of publications, and their clinical relevance could increase in the future. The largest contribution came from the United States followed closely by German-speaking countries such as Germany and Switzerland. Although most of the research comes from the top 10 contributing countries, there is a need for improved international collaboration to solve FRI problems. The most influential scholars in the field come from the United States and Europe. This review also found a positive correlation between citation counts and Research Interest Scores, while the Altmetric Score was found to be negatively correlated with highly cited FRI articles. Finally, we can see that Twitter was the most popular social networking tool among researchers within the social networks analyzed.

## Figures and Tables

**Table 1:** List of the top 10 journals.

Source Title	Number of Publications	Percentage	Impact Factor in 2021	Type of Journal
*Injury—International Journal of the Care of the Injured*	18	13.7%	2.586	Orthopedic Journal
*Journal of Orthopaedic Research*	9	6.9%	3.494	Orthopedic Journal
*Journal of Orthopaedic Trauma*	6	4.6%	2.512	Orthopedic Journal
*Archives of Orthopaedic and Trauma Surgery*	6	4.6%	3.067	Orthopedic Journal
*Bone & Joint Journal*	5	3.8%	5.082	Orthopedic Journal
*Journal of Biomedical Materials Research Part B—Applied Biomaterials* *International Orthopaedics*	4 4	3.1% 3.1%	3.368 3.075	Journal of Biomedical Materials Science Orthopedic Journal
*European Journal of Nuclear Medicine and Molecular Imaging* *Journal of Clinical Medicine*	4 3	3.1% 2.3%	9.236 4.241	Nuclear Medicine Journal Clinical Research
*Biomaterials*	3	2.3%	12.479	Biomaterials Journal

**Table 2:** List of the top 10 most cited articles.

Title	Author, Year	Journal	Total Citations	OA	Type of Article	Level of Evidence
Bacteria antibiotic resistance: New challenges and opportunities for implant-associated orthopedic infections	Li and Webster, 2018 [24]	*Journal of Orthopaedic Research*	268	yes	original	II
Rapid Biofilm Eradication on Bone Implants Using Red Phosphorus and Near-Infrared Light	Tan et al., 2018 [25]	*Advanced materials*	137	no	original	II
Fracture-related infection: A consensus on definition from an international expert group	Metsemakers et al., 2018 [11]	*Injury—International Journal of the Care of the Injured*	104		original	V
Selective laser melting porous metallic implants with immobilized silver nanoparticles kill and prevent biofilm formation by methicillin-resistant Staphylococcus aureus	van Hengel et al., 2017 [26]	*Biomaterials*	97		original	II
Orthopaedic biofilm infections	Zimmerli and Sendi, 2017 [27]	*APMIS*	90	yes	review	II
Antimicrobial coated implants in trauma and orthopaedics—A clinical review and risk–benefit analysis	Alt, 2017 [28]	*Injury—International Journal of the Care of the Injured*	51	no	review	III
Remote eradication of biofilm on titanium implant via near-infrared light triggered photothermal/photodynamic therapy strategy	Yuan et al., 2019 [29]	*Biomaterials*	49	no	original	IV
The effect of local antibiotic prophylaxis when treating open limb fractures: A systematic review and meta-analysis	Morgenstern et al., 2018 [30]	*Bone & Joint Research*	39	yes	systematic review	I
Definition of infection after fracture fixation: A systematic review of randomized controlled trials to evaluate current practice	Metsemakers et al., 2018 [8]	*Injury—International Journal of the Care of the Injured*	31	yes	systematic review	I
The value of quantitative histology in the diagnosis of fracture-related infection	Morgenstern et al., 2018 [31]	*Bone & Joint Journal*	29	yes	original	I

## Data Availability

The data that support the findings of this study are available on request from the corresponding author (MR).
